# Laparoscopy-assisted versus open surgery for multiple colorectal cancers with two anastomoses: a cohort study

**DOI:** 10.1186/s40064-016-1948-4

**Published:** 2016-03-08

**Authors:** Hiroaki Nozawa, Soichiro Ishihara, Koji Murono, Koji Yasuda, Kensuke Otani, Takeshi Nishikawa, Toshiaki Tanaka, Tomomichi Kiyomatsu, Keisuke Hata, Kazushige Kawai, Hironori Yamaguchi, Toshiaki Watanabe

**Affiliations:** Department of Surgical Oncology, The University of Tokyo, 7-3-1 Hongo, Bunkyo-ku, Tokyo, 113-8655 Japan

**Keywords:** Multiple colorectal cancers, Laparoscopic surgery, Combined resections, Two anastomoses

## Abstract

Laparoscopy-assisted surgery has been widely accepted in the treatment of colorectal cancer. The aim of the present study was to investigate the feasibility and outcomes of laparoscopy-assisted combined resection for multiple colorectal cancers in comparison to open surgery. We retrospectively reviewed patients with synchronous multiple colorectal cancers who underwent combined resection resulting in two anastomotic sites by either open or laparoscopy-assisted surgery in the University of Tokyo Hospital between April 2005 and March 2015. Nine patients underwent laparoscopic surgery using five ports, whereas 16 underwent open surgery. Blood loss was less (median 65 vs 295 mL, p = 0.0015), but the operative time was longer (median 429 vs 310 min, p = 0.09) in the laparoscopic surgery group than in the open surgery group. No intergroup difference was observed in the number of lymph nodes retrieved (median 32 vs 27, p = 0.50). The frequency of clinically significant postoperative complications was also similar between the two groups. Our results suggest that laparoscopy-assisted combined resection is an acceptable alternative to open surgery for multiple colorectal cancers.

## Background

Colorectal cancer (CRC) is one of the most prevalent malignancies in the world and carries the second highest mortality rate (Jemal et al. 2011). CRCs are more likely to develop simultaneously with malignant neoplasms in other segments than other gastrointestinal cancers. The recent reported incidence of synchronous CRCs ranged from 1 to 8 % (Piñol et al. 2004; van Leersum et al. 2014; Lam et al. 2014). These cancers often require the resection of multiple segments when lesions are located separately from one another.

Laparoscopic surgery has become the standard for CRC in many institutions and has considerable benefits such as being less invasiveness and achieving better cosmetic outcomes. Furthermore, previous randomized studies demonstrated that a laparoscopic approach was technically feasible and similar or even superior to open surgery in terms of short- and long-term outcomes in CRC (Weeks et al. 2002; Guillou et al. 2005; Lacy et al. 2002; Lujan et al. 2009; Colon Cancer Laparoscopic or Open Resection Study Group et al. 2009; Jayne et al. 2010; van der Pas et al. 2013). However, the feasibility of laparoscopic combined resection of two segments for multiple CRCs has not yet been fully investigated.

We herein analyzed the surgical outcomes of patients with multiple CRCs who underwent laparoscopy-assisted resection with two anastomotic sites in comparison to those who underwent combined resection by open laparotomy.

## Methods

### Patients and data collection

We retrospectively analyzed patients diagnosed with double primary CRCs who underwent surgical resection resulting in two anastomoses in our department between April 2005 and May 2015. All patient data regarding age, sex, Eastern Cooperative Oncology Group (ECOG) performance status (PS), body mass index (BMI), comorbidities, and serum levels of hemoglobin, albumin, carcinoembryonic antigen (CEA), and carbohydrate antigen (CA) 19-9 were reviewed. Patients were classified into either a laparoscopic (LAP) or open laparotomy (OP) group by the surgical approach. Surgical procedures including the extent of lymphadenectomy and temporary stoma, operative time, estimated blood loss, intraoperative blood transfusion, total number of lymph nodes harvested, and complications occurring within 30 days of surgery were documented. As well as obstructive lesion which a colonoscope could not pass through preoperatively, the pathological parameters of each primary tumor, namely, the location, histological grade, maximum tumor diameter, depth, extent of regional lymph node metastases, and final stage were noted. The extent of lymph node dissection was defined previously (Watanabe et al. 2015). Postoperative complications were graded by the Clavien–Dindo classification (Dindo et al. 2004). The final stage was determined according to the classification established by the American Joint Committee on Cancer (Edge et al. 2010).

### Evaluation of vascular branching

Understanding vascular branching is an important process in performing combined resections of separate colorectal segments. In order to evaluate the branching patterns of the superior and inferior mesenteric arteries, patients routinely underwent preoperative three-dimensional-computed tomography angiography. We always confirmed that feeding vessels for tumor-free segments could be preserved in preoperative simulation or during surgery; otherwise other surgical procedures such as subtotal colectomy were considered.

### Surgery

Among multiple CRCs, mobilization of the cancer-bearing segment and lymphadenectomy were initially performed for more advanced-stage lesions. For each tumor-bearing segment, standard surgical resection includes proximal ligation of the feeding vascular pedicle, with en bloc lymph node retrieval of associated draining lymph nodes. In all cases, surgery was performed by well-trained laparoscopic surgeons.

**Fig. 1:**
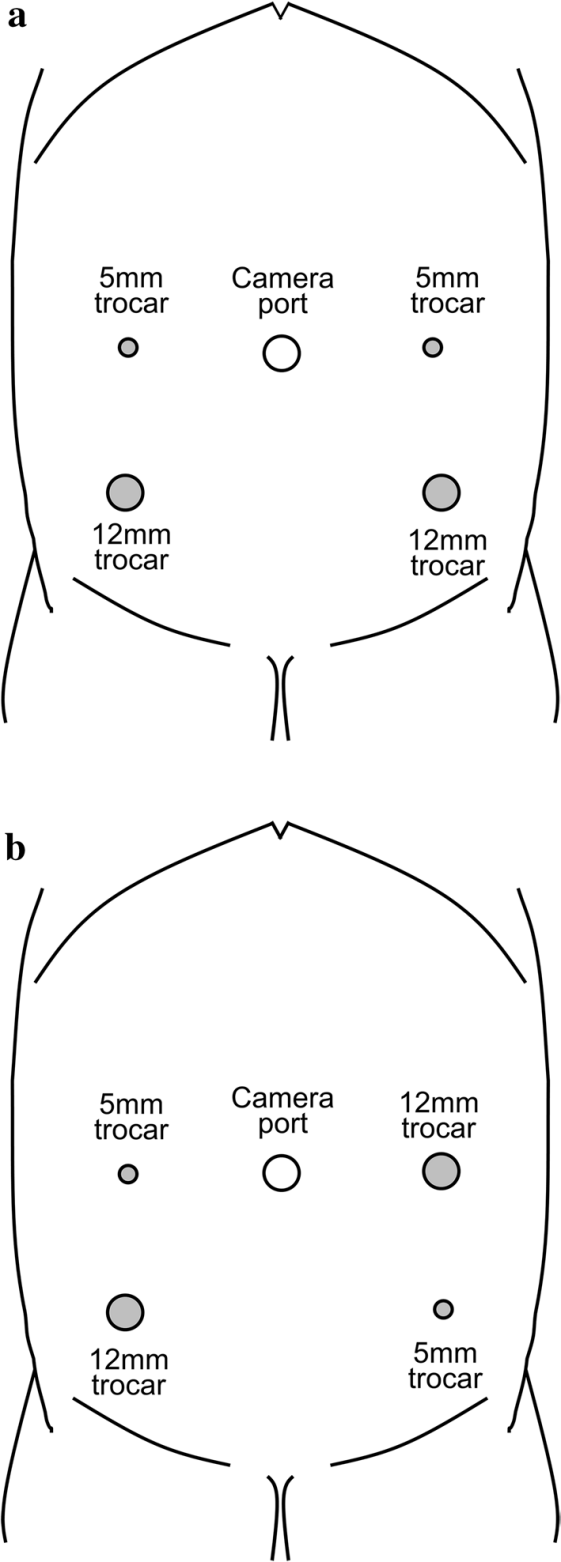
**a** Schematic positions of the camera port and trocars for laparoscopic resection of sigmoid, rectosigmoid, or rectal cancer **b** Schematic positions of the camera port and trocars for laparoscopic resection of cancer in another location

In laparoscopy-assisted surgery, pneumoperitoneum was created through a 12-mm camera trocar at the umbilicus. In laparoscopy-assisted surgery, pneumoperitoneum was created through a 12-mm camera trocar at the umbilicus. An additional four trocars (two 12-mm and two 5-mm) enabled access to the abdominal cavity. We generally positioned a 12-mm trocar in the right lower quadrant and a second 12-mm trocar in the left lower quadrant when either lesion was located in the sigmoid or distal bowel (Fig. 1a). In other situations, the second 12-mm trocar was placed in the left upper quadrant (Fig. 1b).

Regarding right-sided lesions (proximal to the middle of the transverse colon), after proximal ligation of the tumor-feeding vessels (the ileocolic, right colic, and/or right branch of the middle colic vessels), mobilization of the right colon and takedown of the hepatic flexure were performed intracorporeally together with dissection of the draining lymph nodes from a medial-to-lateral direction. Mobilization was completed by cutting the peritoneum from the lateral side.

Regarding left-sided lesions (distal to the middle of the transverse colon and descending colon), the left colon and splenic flexure was mobilized after ligation of the left colic (and the left branches of the middle colic) vessels, similar to right-sided lesions.

Regarding sigmoid colon and rectosigmoid lesions, the left colon was mobilized from a medial-to-lateral direction and intracorporeal high ligation was performed at or near the roots of the inferior mesenteric vessels. The inferior mesenteric artery was ligated under the level of the bifurcation of the left colic artery for rectal lesions. After rectal mobilization in the layer targeted for total mesorectal excision with autonomic nerve preservation, the bowel distal to the cancer was transected intracorporeally by linear staplers.

The two mobilized bowel segments were exteriorized under wound protection through a 3.5-cm incision longitudinally extended at the camera-port site. The mini-incision was extended further when necessary. After division of the marginal vessels, both specimens were removed.

Ileocolostomy and colocolostomy were extracorporeally achieved by stapled side-to-end or functional end-to-end anastomosis. In coloproctostomy, we placed the anvil of a circular stapler in the proximal sigmoid, and the mini-incision was then covered to re-establish pneumoperitoneum. An anastomosis was intracorporeally performed using the double stapling technique under laparoscopic visualization.

In open surgery, a middle to lower midline incision was made dependently on the locations of CRCs. The mobilization of colonic segments was conducted from a lateral-to-medial direction. Mobilization of the rectum and mesorectum, the ligation of tumor-feeding vessels, and anastomoses were performed similarly to the laparoscopic approach.

Each surgeon made a final decision regarding whether a temporary stoma needed to be placed based on the distal anastomosis level from the anal verge as well as the patient’s condition.

### Statistical analysis

Differences between patient characteristics in the open and laparoscopic groups were evaluated using the Chi square test for categorical variables and the Mann–Whitney U test for continuous variables. A p value of less than 0.05 was considered to denote significance. All statistical analyses were performed using the statistical software program JMP version 11.0.2 (SAS Institute Inc., Cary, NC, USA).

## Results

### Characteristics of patients

The LAP group comprised nine patients (seven men and median age 71 years old), and the OP 16 patients (12 men and median age 71 years old). The baseline characteristics of the patients are shown in Table 1. Eighty percent of patients had an ECOG PS of 0, and the median BMI was 22. Approximately two-thirds had comorbid illnesses; more patients had nephrological disorders in the LAP group (44 %) than in the OP (6 %, p = 0.04). Median albumin and hemoglobin levels were 3.5 and 11.4 g/dL, respectively, without significant intergroup differences. Elevated CEA and CA19-9 levels in the serum were observed in more patients in the OP (62 and 31 %) than in the LAP groups (22 and 0 %), but these differences were not significant.

**Table 1:** Clinical profile of patients

	LAP (n = 9)	OP (n = 16)	Total (n = 25)	*p* value
Gender				
Male	7 (78 %)	11 (69 %)	18 (72 %)	1.00
Female	2 (22 %)	5 (31 %)	7 (28 %)	
Age (years)				
Median (range)	71 (61–81)	71 (50–80)	71 (50–81)	0.54
PS				
0	8 (89 %)	12 (75 %)	20 (80 %)	0.62
1 or more	1 (11 %)	4 (25 %)	5 (20 %)	
BMI (kg/m^2^)				
Median (range)	23 (19–26)	22 (16–27)	22 (16–27)	0.33
Comorbidity^a^				
None	2 (22 %)	6 (38 %)	8 (32 %)	0.66
Cardiovascular	5 (56 %)	5 (31 %)	10 (40 %)	0.44
Diabetes	2 (22 %)	3 (19 %)	5 (20 %)	1.00
Pulmonary	0 (0 %)	2 (13 %)	2 (8 %)	0.52
Hepatic	0 (0 %)	1 (6 %)	1 (4 %)	1.00
Nephrological	4 (44 %)	1 (6 %)	5 (20 %)	0.04
Neurological	2 (22 %)	2 (13 %)	4 (16 %)	0.60
Hb (g/dL)				
Median (range)	11.4 (9.7–14.3)	11.3 (8.4–13.8)	11.4 (8.4–14.3)	0.52
Alb (g/dL)				
Median (range)	3.5 (3.1–4.3)	3.7 (1.9–4.2)	3.5 (1.9–4.3)	0.56
CEA (ng/mL)				
Elevated	2 (22 %)	10 (62 %)	12 (48 %)	0.10
CA19-9 (U/mL)				
Elevated	0 (0 %)	5 (31 %)	5 (20 %)	0.12

*Hb* hemoglobin, *Alb* albumin, *CEA* carcinoembryonic antigen, *CA19*-*9* carbohydrate antigen 19-9

^a^Multiple comorbidies in some cases

### Feature of CRCs

Most patients had double CRCs, except for five patients with triple CRCs and one with quadruple CRCs. These six patients underwent open surgery for removal of all lesions in two segments. Table 2 summarizes the findings of CRCs. Obstructive cancers were found in two patients in the LAP group and six in the OP group. CRCs were most frequently located in the ascending and sigmoid colon (both 25 %), followed by the transverse colon and rectum (both 18 %). No cancer was detected in the descending colon. The median size of tumors was 30 mm in the LAP group and 33 mm in the OP group (p = 0.20). All CRCs in the LAP group showed differentiated histology, whereas 13 % of lesions in the OP group contained other histological components such as poorly differentiated adenocarcinoma and mucinous carcinoma (p = 0.16). The most frequent depth of CRCs was T3 (39 %), followed by Tis/T1 (36 %). T4 tumors were only found in the OP group (13 %). Locoregional lymph nodes were metastasized in 12 % of CRCs in the LAP group and 22 % in the OP group (p = 0.96). As the most frequent stage, 56 % of CRCs were diagnosed as Stage I in the LAP group, whereas 42 % of CRCs were Stage II in the OP group. The disproportion in the final stage between the LAP and OP was not significant (p = 0.44).

**Table 2:** Findings of multiple CRCs

	LAP (n = 18)	OP (n = 38)	Total (n = 56)	*p* value
Obstruction				
Yes	2 (22 %)	6 (16 %)	8 (14 %)	1.00
Location				
Cecum	3 (16 %)	5 (13 %)	8 (14 %)	0.86
Ascending	5 (28 %)	9 (24 %)	14 (25 %)	
Transverse	2 (22 %)	8 (21 %)	10 (18 %)	
Descending	0 (0 %)	0 (0 %)	0 (0 %)	
Sigmoid	3 (16 %)	11 (29 %)	14 (25 %)	
Rectum^a^	5 (28 %)	5 (13 %)	10 (18 %)	
Tumer size (mm)^b^				
Median (range)	30 (0–88)	33 (4–48)	30 (0–88)	0.20
Histological type				
Diff. adenoca.	18 (100 %)	33 (87 %)	51 (91 %)	0.16
Others	0 (0 %)	5 (13 %)	5 (9 %)	
T				
Tis/T1	7 (39 %)	13 (34 %)	20 (36 %)	0.16
T2	6 (33 %)	3 (8 %)	9 (16 %)	
T3	5 (28 %)	17 (45 %)	22 (39 %)	
T4	0 (0 %)	5 (13 %)	5 (9 %)	
N				
N0	16 (88 %)	30 (78 %)	46 (82 %)	0.96
N1	1 (6 %)	4 (11 %)	5 (9 %)	
N2	1 (6 %)	4 (11 %)	5 (9 %)	
Stage				
0	2 (11 %)	4 (11 %)	6 (11 %)	0.44
I	10 (56 %)	11 (29 %)	21 (37 %)	
II	4 (22 %)	16 (42 %)	20 (36 %)	
III	2 (11 %)	7 (18 %)	9 (16 %)	

*Diff. adenoca.* differentiated adenocarcinoma

^a^Including rectosigmoid

^b^Including lesions that showed a pathologically complete response to preoperative chemoradiation therapy

### Surgery and perioperative outcomes

In the LAP group, all patients underwent the complete removal of multiple CRCs without conversion to open laparotomy and did not require additional trocars. Table 3 depicts details of the surgical procedures for each CRC. The distribution of resected segments showed similar patterns between the LAP and OP groups; the proximal segment was resected mostly by right-sided colectomy, while the distal was predominantly removed by sigmoid colectomy or anterior resection of the rectum. There was no obvious intergroup difference in the extent of lymphadenectomy. All patients underwent R0 resection.

**Table 3:** Details of surgery for each cancer

	LAP (n = 18)	OP (n = 32)	Total (n = 50)	*p* value
Surgical procedure				
Rt-sided colectomy	9 (50 %)	13 (41 %)	22 (44 %)	0.84
Transverse colectomy	0 (0 %)	4 (12 %)	4 (8 %)	
Lt-sided colectomy	1 (5 %)	2 (6 %)	3 (6 %)	
Sigmoid colectomy	3 (17 %)	7 (22 %)	10 (20 %)	
Anterior resection	5 (28 %)	6 (19 %)	11 (22 %)	
Extent of lymphade-nectomy				
D1	1 (6 %)	9 (28 %)	10 (20 %)	0.29
D2	6 (33 %)	6 (19 %)	12 (24 %)	
D3	11 (61 %)	17 (53 %)	28 (56 %)	

*Rt*-*sided* right-sided, *Lt*-*sided* left-sided

Table 4 summarizes LAP and OP surgery for individual patients. Covering ileostomy was created in one patient in the LAP group. Operative time was slightly longer in the LAP group (median 429 min) than in the OP group (median 310 min, p = 0.09), whereas the estimated volume of blood loss was markedly smaller in the LAP group (median 65 mL) than in the OP group (median 295 mL, p = 0.0015). Only two patients (13 %) in the OP group received an intraoperative blood transfusion. No significant difference was observed in the total number of harvested lymph nodes between the LAP and OP groups (median 32 and 27, p = 0.50).

**Table 4:** Overview of surgery for individual patients

	LAP (n = 9)	OP (n = 16)	Total (n = 25)	*p* value
Temporary stoma				
Yes	1 (11 %)	0 (0 %)	1 (4 %)	0.36
Operative time (min)				
Median (range)	429 (227–655)	310 (171–461)	330 (171–655)	0.09
Estimated blood loss (mL)				
Median (range)	65 (30–210)	295 (130–930)	210 (30–930)	0.0015
Blood transfusion				
Yes	0 (0 %)	2 (13 %)	2 (8 %)	0.52
Number of lymph nodes harvested^a^				
Median (range)	32 (15–60)	27 (11–147)	29 (11–147)	0.50
Complications				
None/grade 1	6 (67 %)	12 (75 %)	18 (72 %)	0.67
Grade 2 or more	3 (33 %)	4 (25 %)	7 (28 %)	

^a^Counting together for multiple cancers

Postoperative complications (Clavien–Dindo classification grade 2 or greater) occurred in 33 % in the LAP group and 25 % in the OP group (p = 0.67).

## Discussion

LAP and OP for CRCs have been compared in many randomized prospective studies in various countries; their findings were summarized as follows: LAP is characterized by (1) a longer operative time, (2) less blood loss, (3) faster postoperative recovery and shorter length of stay, (4) fewer complications, e.g. wound infection, and (5) similar oncological outcomes to OP (Weeks et al. 2002; Guillou et al. 2005; Lacy et al. 2002; Lujan et al. 2009; Colon Cancer Laparoscopic or Open Resection Study Group et al. 2009; Jayne et al. 2010; van der Pas et al. 2013). These findings were obtained with respect to surgical resection of a single segment.

In the last decade, the safety and feasibility of laparoscopic resection of two colorectal segments have occasionally been documented in case reports (Jafari Giv and Ho 2007; Tan et al. 2012; Inada et al. 2014; Fang et al. 2015). Inada et al. previously described 11 successful cases of laparoscopic combined resection for synchronous CRCs by similar procedures to ours including a patient undergoing rectal amputation (Inada et al. 2014). However, none of these case reports compared their cases with the corresponding open surgical procedures exclusively for CRCs. To the best of our knowledge, the current study is the first to have analyzed LAP and OP surgery resulting in two anastomoses for multiple CRCs.

None of our LAP cohort required conversion to open surgery or additional trocars. Moreover, we found a marked reduction in blood loss in the LAP group. Another benefit of LAP for multiple CRCs appeared to be minimized wound lengths, although we had no prospective data on the total length of incisions in our cases. Open surgery to resect two colorectal segments may inevitably have needed a markedly longer incision than for a single segment. On the other hand, operative time was 119 min longer in the LAP group than in the OP group. This difference was more than the theoretical time, i.e. twice as long as the gap in operative duration between LAP and OP for resection of a single segment that reportedly ranged from 22 to 52 min (Guillou et al. 2005; Lacy et al. 2002; Lujan et al. 2009; Colon Cancer Laparoscopic or Open Resection Study Group et al. 2009; van der Pas et al. 2013). It may have mainly been caused by difficulties in the mobilization of separate segments due to the fixed positions of the five trocars. The estimated blood loss, operative time, and rate of postoperative complications in our LAP group were consistent with the previous findings of Inada et al. (15–441 mL, 235–467 min, and 22 %, respectively) (Inada et al. 2014).

With regard to oncological aspects, R0 resection could be achieved in 100 %. Moreover draining lymph nodes were similarly harvested in both groups. Due to slightly higher rate of more aggressive CRCs in the OP group and the shorter duration of follow-up in the LAP, we did not consider it suitable to compare the long-term results of combined resection between the LAP and OP groups in our study. Prospective randomized controlled trials will be required to evaluate the oncological outcomes of LAP for multiple CRCs.

Our study had several other limitations. Our cohort comprised a limited number of patients. Due to its retrospective nature, potential biases existed in the selection of LAP and OP approaches. For example, T4 lesions were only operated on via an OP approach. Surgeons as well as surgical procedures were not matched between the two groups. Since the OP group included rather old cases before the concept of fast-track pathways, it was difficult to compare postoperative recovery and the length of the hospital stay between the LAP and OP groups.

## Conclusions

We herein demonstrated that laparoscopy-assisted combined resection for multiple CRCs with two anastomotic sites were safe and feasible, if the surgeon is well trained in colorectal surgery. The relevance of laparoscopic surgery for multiple CRCs from an oncological aspect needs to be addressed by studies with larger patient numbers and a longer follow-up.

## Abbreviations

CRC: colorectal cancer
ECOG: Eastern Cooperative Oncology Group
PS: performance status
BMI: body mass index
CEA: carcinoembryonic antigen
CA19-9: carbohydrate antigen 19-9

## References

[CR1] Buunen M, Veldkamp R, Colon Cancer Laparoscopic or Open Resection Study Group (2009). Survival after laparoscopic surgery versus open surgery for colon cancer: long-term outcome of a randomised clinical trial. Lancet Oncol.

[CR2] Dindo D, Demartines N, Clavien PA (2004). Classification of surgical complications: a new proposal with evaluation in a cohort of 6336 patients and results of a survey. Ann Surg.

[CR3] Edge S, Byrd D, Compton C (2010). AJCC cancer staging manual.

[CR4] Fang JF, Zheng ZH, Huang Y (2015). Laparoscopy-assisted combined resection for synchronous gastrointestinal multiple primary cancers. Int J Surg.

[CR5] Guillou PJ, Quirke P, Thorpe H (2005). Short-term endpoints of conventional versus laparoscopic-assisted surgery in patients with colorectal cancer (MRC CLASICC trial): multicentre, randomised controlled trial. Lancet.

[CR6] Inada R, Yamamoto S, Takawa M (2014). Laparoscopic resection of synchronous colorectal cancers in separate specimens. Asian J Endosc Surg.

[CR7] Jafari Giv M, Ho YH (2007). Concurrent laparoscopic right hemicolectomy and ultra-low anterior resection with colonic J-pouch anal anastomosis for synchronous carcinoma. Tech Coloproctol.

[CR8] Jayne DG, Thorpe HC, Copeland J (2010). Five-year follow-up of the Medical Research Council CLASICC trial of laparoscopically assisted versus open surgery for colorectal cancer. Br J Surg.

[CR9] Jemal A, Bray F, Center MM (2011). Global cancer statistics. CA Cancer J Clin.

[CR10] Lacy AM, García-Valdecasas JC, Delgado S (2002). Laparoscopy-assisted colectomy versus open colectomy for treatment of non-metastatic colon cancer: a randomised trial. Lancet.

[CR11] Lam AK, Chan SS, Leung M (2014). Synchronous colorectal cancer: clinical, pathological and molecular implications. World J Gastroenterol.

[CR12] Lujan J, Valero G, Hernandez Q (2009). Randomized clinical trial comparing laparoscopic and open surgery in patients with rectal cancer. Br J Surg.

[CR13] Piñol V, Andreu M, Castells A (2004). Synchronous colorectal neoplasms in patients with colorectal cancer: predisposing individual and familial factors. Dis Colon Rectum.

[CR14] Tan WS, Quah HM, Eu KW (2012). Combined laparoscopic anterior resection and right hemicolectomy for synchronous colorectal tumours: how to retrieve both specimens at the same time through a transverse incision. Surgeon.

[CR15] van der Pas MH, Haglind E, Cuesta MA (2013). Laparoscopic versus open surgery for rectal cancer (COLOR II): short-term outcomes of a randomised, phase 3 trial. Lancet Oncol.

[CR16] van Leersum NJ, Aalbers AG, Snijders HS (2014). Synchronous colorectal carcinoma: a risk factor in colorectal cancer surgery. Dis Colon Rectum.

[CR17] Watanabe T, Itabashi M, Shimada Y (2015). Japanese Society for Cancer of the Colon and Rectum (JSCCR) Guidelines 2014 for treatment of colorectal cancer. Int J Clin Oncol.

[CR18] Weeks JC, Nelson H, Gelber S (2002). Short-term quality-of-life outcomes following laparoscopic-assisted colectomy vs open colectomy for colon cancer: a randomized trial. JAMA.

